# When Insult Is Added to Injury: Cross Talk between ILCs and Intestinal Epithelium in IBD

**DOI:** 10.1155/2016/9765238

**Published:** 2016-08-04

**Authors:** Esmé van der Gracht, Sonja Zahner, Mitchell Kronenberg

**Affiliations:** ^1^Division of Developmental Immunology, La Jolla Institute for Allergy and Immunology, 9420 Athena Circle, La Jolla, CA 92037, USA; ^2^Department of Immunohematology and Blood Transfusion, Leiden University Medical Center, Albinusdreef 2, 2333 ZA Leiden, Netherlands; ^3^Division of Biological Sciences, University of California San Diego, 9500 Gilman Drive, La Jolla, CA 92093, USA

## Abstract

Inflammatory bowel disease (IBD) is characterized by an impairment of the integrity of the mucosal epithelial barrier, which causes exacerbated inflammation of the intestine. The intestinal barrier is formed by different specialized epithelial cells, which separate the intestinal lumen from the lamina propria. In addition to its crucial role in protecting the body from invading pathogens, the intestinal epithelium contributes to intestinal homeostasis by its biochemical properties and communication to underlying immune cells. Innate lymphoid cells (ILCs) are a recently described population of lymphocytes that have been implicated in both mucosal homeostasis and inflammation. Recent findings indicate a critical feedback loop in which damaged epithelium activates these innate immune cells to restore epithelial barrier function. This review will focus on the signalling pathways between damaged epithelium and ILCs involved in repair of the epithelial barrier and tissue homeostasis and the relationship of these processes with the control of IBD.

## 1. Introduction

Inflammatory bowel disease (IBD) comprises a group of intestinal inflammatory conditions that are characterized by an exacerbated chronic inflammation of the gastrointestinal tract. An estimated 1,171,000 Americans are currently diagnosed with IBD and there is increasing prevalence in different regions around the world [1, 2]. IBD includes two related chronic and relapsing conditions, ulcerative colitis and Crohn's disease, that have a severe impact on quality of life. Symptoms of IBD often include abdominal pain, diarrhea, and rectal bleeding, as well as systemic symptoms of weight loss, fever, and fatigue. Periods of remission, in which patients have no symptoms, are alternated by clinical relapses, during which symptoms worsen. These symptoms are the consequence of a dysregulated immune system, of which the origin remains uncertain. It has been suggested that IBD is caused by an abnormal immune reaction to normal constituents of the mucosal microbiota, but genetics and environmental factors also appear to influence disease risk. In addition, changes in the composition of the intestinal microbiota were found to be important environmental factors in IBD pathology [3]. These factors can induce an overactive immune response that damages the mucosal barrier.

In both Crohn's disease and ulcerative colitis, an impairment of the integrity of the mucosal epithelial barrier is observed. The intestinal epithelium of the colon is essential for the absorption of water, but it also has a crucial role as a protective physical barrier by separating the intestinal lumen from the lamina propria. The pathogenesis of IBD differs between ulcerative colitis and Crohn's disease in location and extent of inflammation. While Crohn's disease affects the small bowel and colon, with discontinuous ulceration and full thickness bowel wall inflammation, ulcerative colitis primarily affects the colon, with a continuous inflammation of the mucosa nearly always involving the rectum. In Crohn's disease, deep ulcers can cause an infection outside the bowel wall, which can spread to the skin or other organs and which can form inflammatory connections called fistula.

In mice, experimental colitis can be induced under several experimental conditions. For example, mice possessing genetic deficiencies in both TGF*β*RII and IL-10R2 (dnKO mice) develop a disease similar to human ulcerative colitis [4]. However, colitis can also be induced in mice by exposure to* Helicobacter hepaticus* or* Citrobacter rodentium*, by treatment with anti-CD40 monoclonal antibodies (mAbs), by transfer of CD4^+^ T cells depleted of regulatory T cells into immune deficient mice, or by chemical damage to the colon epithelium, for example, by treatment with dextran sodium sulfate (DSS). Depending on the protocol and dosage of DSS administration, mice can be studied in either a chronic or relapsing colitis model. Both models have similarities with human ulcerative colitis and seem to be driven predominantly by innate immune cells, as disease can be elicited in RAG deficient mice that lack B and T lymphocytes. The exact mechanism by which DSS induces intestinal inflammation remains unknown, but it has been suggested that DSS induces tight junction (TJ) disruption, supporting the hypothesis that barrier dysfunction can occur prior to the onset of mucosal inflammation [5]. DSS-induced colitis is therefore an appropriate model to study the role of candidate genes in colitis promoted by a loss of the epithelial integrity. Consistent with the importance of barrier function, a recent paper showed that increased paracellular permeability for bacterial products, due to claudin 7 deficiency with resulting effects on tight junctions, played a critical role in initiating colonic inflammation [6]. IBD patients also demonstrated increased paracellular permeability with TJ abnormalities. In addition to increased TJ permeability, alterations such as induced epithelial cell death are associated with IBD, and loss of IEC integrity was seen in IBD patients by confocal endomicroscopy before clinical relapse of the disease [7]. However, it should be noted that healthy relatives from IBD patients also showed increased intestinal permeability [8]. Thus, it is unlikely that moderate barrier defects alone are sufficient to cause IBD.

Epithelial barrier integrity is critical to maintain a balance between a symbiosis with the microbiota on the one hand and preventing pathogenic microbes from entering the body on the other hand. The recognition of pathogen-derived and damage-associated molecules upon disruption of the epithelial lining, for example, recognition via toll-like receptors (TLR) expressed by epithelial cells and innate immune cells residing in the epithelium and lamina propria, initiates the production of inflammatory chemokines and cytokines [9]. This recruits innate and adaptive immune cells, which ideally clear the pathogens that entered the lamina propria and help in restoring the epithelial barrier. In order to enable epithelial barrier restoration and reinstate homeostatic conditions, the immune response needs to be downregulated to avoid excessive and unnecessary tissue damage [10]. However, if the pathogen is not sufficiently cleared, or the immune response is not successfully downregulated, chronic inflammation and tissue damage are the consequence.

Communication between mucosal innate immune cells of the myeloid lineage, such as neutrophils, monocytes, macrophages, and antigen-presenting dendritic cells, and the epithelium plays a critical role in regulating gut homeostasis and intestinal inflammation. The recent discovery of innate lymphoid cells (ILCs) added a new player to the field of immunology. ILCs have been implicated in regulating immune homeostasis and inflammatory responses in several instances, such as allergies and bacterial infections [11–13]. Although the ILC population in the intestine is only a fraction of the total lymphocytes, they can contribute to IBD pathology by producing large amounts of cytokines in response to signals from microbes, myeloid cells, and/or epithelial cells [14–16]. Epithelial damage in IBD leads to an altered interaction with the underlying immune cells. Recent findings indicate a critical feedback loop in which damaged epithelium activates ILCs to restore epithelial barrier function [17–20]. Understanding the cross talk between these innate immune cells and the intestinal epithelium in IBD will provide more insight into dysregulation of intestinal homeostasis and IBD pathogenesis.

## 2. Epithelial Barrier Dysfunction in IBD

The intestinal epithelium is a monolayer of specialized intestinal epithelial cells (IECs), which differentiate from epithelial stem cells into different cell lineages that form crypt and villi structures, including absorptive enterocytes, enteroendocrine cells, mucus-secreting goblet cells, and tuft cells [21]. IECs proliferate in the crypt and migrate to the apical zone while differentiating; mature enterocytes move up the villi and are constantly shed from the villus tip. The continual turnover of epithelial cells helps to maintain barrier function by replenishing the epithelial layer with fresh cells and thereby enables quick repair.

### 2.1. Epithelial Injury

Damage of the epithelial barrier could be caused by an infection, an exacerbated immune response to normal constituents of the intestine, or a reaction to noninfectious agents. Proinflammatory cytokines from lamina propria immune cells are produced in response to various signals to fight infections and maintain homeostasis. Key antigen-presenting cells (APCs) such as dendritic cells and macrophages are activated in response to molecules from microbes and produce proinflammatory cytokines such as IL-1*β*, IL-6, IL-18, and tumor necrosis factor (TNF). Different cytokines contribute to the resistance to infections; however, exacerbated cytokine production can lead to pathology. Excessive production of cytokines can cause epithelial barrier dysfunction by altering tight junction and inducing IEC apoptosis and pathological cell shedding. For example, TNF can induce pathological intestinal epithelial shedding by initiating epithelial apoptosis and IL-6 is able to modulate intestinal epithelial tight junction permeability [22, 23]. The regulation of the rate of proliferation and cell death of IECs usually ensures quick and efficient repair of mechanical damage to the epithelium [24]. However, a recent study showed a previously unidentified epithelial-intrinsic alteration of cell death in tissue from IBD patients. Disease-specific alterations in IECs with impaired Rho-A signalling in IBD patients were found and impaired Rho-A signalling in mice caused cytoskeletal alterations and altered cell shedding, which in turn led to intestinal inflammation [25].

In addition to dysregulated cytokine production, genetic and environmental factors can contribute to intestinal barrier loss. Two well-known susceptibility genes for IBD development are NOD2/CARD15, which is involved in bacterial peptidoglycan recognition, and ATG16L1, which plays a role in autophagy, a process related to processing and presentation of bacterial components [26–29]. It has been found that risk alleles of NOD2 and ATG16L1 are associated with shifts in microbial compositions, associated with development of IBD [3]. Thus, one of the important environmental factors in IBD pathology is a change in the composition of intestinal microbiota. Environmental triggers for IBD are thought to include stress, smoking, diet, and environmental pollution. The underlying mechanisms and their respective contribution to the development of IBD in some people but not in others remain to be elucidated. While environmental factors are believed to contribute to mucosal permeability in IBD, interestingly, permeability is also increased in a proportion of healthy spouses of CD patients [30]. This suggests that an accumulation of multiple factors is necessary to cause IBD.

### 2.2. Response after Injury

In response to damage, epithelial cells secrete various signals of danger. Levels of damage-associated molecular patterns (DAMPs) or alarmins, including IL-1*α* and IL-33, are increased in intestinal tissue from IBD patients [31, 32]. These DAMPs are intracellular proteins under homeostatic conditions but are released upon danger signals, such as tissue damage from different cell types in the intestine, including IECs. Activated IECs can produce various cytokines of the IL-1 family including IL-18, IL-33, and IL-37 [32–34]. Whereas IL-18 induces the production of inflammatory cytokines, IL-37 mainly suppresses mucosal innate immune responses and reduces IL-1*β* and TNF production. IL-33, by contrast, suppresses Th1-type cytokine production and induces neutrophil influx, although in some cases IL-33 in combination with other cytokines augments cytokine production.

APCs, but also neutrophils and ILCs, are potent producers of IL-22 upon activation [35, 36]. This in turn leads to the activation of IL-22 receptors on IECs and the production of various cytokines, for example, IL-10, and antimicrobial peptides such as RegIII-*β* and RegIII-*γ* to eliminate bacterial pathogens [37]. IL-22 has recently been shown to promote intestinal stem cell-mediated epithelial regeneration and therefore is considered crucial for the maintenance of epithelial integrity and regeneration after injury [17]. In general, IECs cytokine production and their response to various cytokines are fundamental to mucosal healing [38]. ILCs are activated by and produce cytokines such as IL-6 and TNF, respectively, that not only have a role in epithelial permeability and IEC apoptosis but also are linked to IBD pathogenesis [39–41]. The epithelial response after injury involves different pro- and anti-inflammatory cytokines; the balance of these cytokines seems to be crucial for immune homeostasis and epithelial integrity in IBD.

### 2.3. Epithelial Repair

After injury of the intestinal epithelium, a process called epithelial restitution initiates wound healing. Enterocytes surrounding the damaged epithelium can migrate into the lesion in response to various cytokines such as transforming growth factor alpha (TGF*α*), epidermal growth factor (EGF), IL-1*β*, and interferon-gamma (IFN-*γ*), although other pathways that induce epithelial restitution also exist [42]. In response, epithelial-derived secreted proteins, including EGF and TGF-*β*1, coordinate epithelial repair [43, 44]. Epithelial cells respond to signals such as EGF, IL-6, IL-22, and toll-like receptor (TLR) ligands to initiate cell proliferation and differentiation in order to resurface the injury. IL-6 is a key cytokine involved in intestinal epithelial proliferation and wound repair and inhibition of IL-6 results in impaired wound healing due to decreased epithelial proliferation, but it also induces permeability, as stated above [40]. In addition to IL-6, IL-22 induces proliferation of IECs by activation of the transcription factor signal transducer and activator of transcription 3 (STAT3) [45]. In contrast to IL-6 and IL-22, IL-13 and TNF-*α* seem to be associated with epithelial barrier dysfunction. A study by Heller et al. showed that lamina propria cells from ulcerative colitis patients produced high levels of IL-13, which was found to affect epithelial apoptosis, tight junctions, and restitution velocity [46]. TNF was found to induce iNOS and thereby activates a p53-dependent pathway of IEC apoptosis in chronic ulcerative colitis [41]. Excessive epithelial apoptosis is obviously linked to barrier dysfunction.

Intestinal epithelial cells sense microbes in the gut lumen by pattern recognition receptors (PRR) that include TLRs and nucleotide oligomerization domain-like receptors (NLRs). Functional pathogen recognition is crucial for the generation of an immune response, but TLR signalling has been also shown to promote restoration of damaged epithelia. For example, TLR9-deficient mice have delayed wound repair and are more susceptible to DSS-induced colitis. TLR9-deficiency led to reduced gene expression of hairy enhancer of split 1, an intestinal progenitor cell differentiation factor, and vascular endothelial growth factor (VEGF), a growth factor important for epithelial cell restitution [47].

Recently, IL-36R signalling in IECs, upon intestinal damage, has been linked to the induction of mucosal healing. Defective IL-36 signalling impaired mucosal wound healing and increased susceptibility to DSS-induced colitis. Scheibe et al. found that IL-36R ligands induced proliferation of IECs and their expression of the antimicrobial protein lipocalin 2 [48]. In addition, a study by Medina-Contreras et al. showed that IL-36R deficient mice did not recover from DSS-induced damage [49].

### 2.4. Barrier Dysfunction

An alteration of epithelial permeability is associated with a loss of mucosal homeostasis, as an impairment of barrier function may fail to sufficiently block pathogen entry. Consequently, pathogen derived antigens are thought to modify the communication of IECs with resident mucosal immune cells, leading to signals that recruit inflammatory cells that clear invading microbes. Genome-wide association studies (GWAS) have identified many IBD susceptibility gene loci, including different STAT proteins and IL-2, IFN-*γ*, and IL-10 [50]. Numerous single nucleotide polymorphisms (SNPs) were identified, in coding and noncoding regions, of which the majority were associated with both ulcerative colitis and Crohn's disease. Another study found that IBD risk loci identified by GWAS colocalized with active DNA regulatory elements in intestinal epithelium and immune cells [51]. This suggests that, in addition to variants in protein coding genes, variants in noncoding DNA regulatory regions that are active in intestinal epithelium and immune cells are potentially involved in the pathogenesis of IBD. Recently, the expression pattern of susceptibility genes was found to be tissue specific, demonstrating how these risk loci can contribute to risk of disease through the modulation of a gene expression [52].

Other studies investigated the level of cytokine expression in tissue from patients and found increased colonic mucosal IL-33 in patients with active ulcerative colitis [32], while IL-22 was found to be increased in active Crohn's disease as a response to damage [53]. Both cytokines may be involved in maintaining epithelial integrity and are key examples of the cross talk between ILCs and epithelial cells in IBD and will be discussed further in this review.

## 3. ILC Populations and Their Function

ILCs have recently been identified as important populations of innate immune effectors and are distributed throughout both lymphoid and nonlymphoid organs, including barrier tissues. They are noncirculating cells that lack antigen-specific receptors encoded by rearranging gene segments, but they differentiate from a common lymphoid progenitor. ILCs can be categorized into functionally distinct subsets, based on their expression of transcription factors and cytokine production [54, 55]. Group 1 ILCs (ILC1 cells and NK cells) express T-bet and/or eomesodermin and produce IFN-*γ* and TNF-*α* and are mainly important for the resistance to intracellular pathogens and the response to tumors. Group 2 ILCs are dependent on the transcription factor GATA-3 and produce cytokines such as IL-4, IL-5, IL-9, and IL-13, essential for immune response to helminths. Group 3 ILCs express retinoic acid receptor- (RAR-) related orphan receptor (ROR*γ*t) and produce lymphotoxin *αβ* (LT*α*1*β*2), granulocyte macrophage-colony stimulating factor (GM-CSF), TNF-*α*, IFN-*γ*, IL-17A, IL-17F, and/or IL-22 and are involved in eliminating extracellular pathogens and restoring epithelial integrity. Group 3 ILCs can be subdivided into natural cytotoxicity receptor (NCR) expressing cells, which secrete IL-22, and NCR negative cells, which include lymphoid tissue inducer cells (LTi), that express both IL-22 and IL-17 [36]. All three ILC subsets have been implicated in IBD pathogenesis and its control.

### 3.1. ILCs in Intestine under Homeostatic Conditions

In both small and large intestine, ILC1 are the most abundant ILC population in the intraepithelial compartment. In the lamina propria, however, ILC2 and ILC3 are the dominant populations in the large and small intestines, respectively. Increased numbers of ILC2s are localized in fat-associated lymphoid clusters in the intestinal mesentery, while ILC3s accumulate in the perifollicular areas of Peyer's patches and near intestinal crypts where they can enter and exit and mobilize elsewhere in the tissue, a process that can be inhibited by blocking GM-CSF [14, 56]. It is still debated in which tissues ILCs are generated from their precursors. It has been suggested that different programs can regulate the migration of ILC subsets to the intestine. A study showed that ILC1 and ILC3 need to undergo a retinoic acid dependent homing receptor switch during their development in gut-associated lymphoid tissue to migrate to intestinal tissue, whereas ILC2 can migrate directly [57]. However, Rudensky's group found that ILCs in the gut can be locally renewed and expanded in response to acute environmental challenges such as helminth infection [58]. This indicates that ILCs may self-renew locally or be generated from precursors in these tissues. It has been suggested that cytokines are responsible for the proliferation and activation of mature ILCs. For instance, ILC2 and ILC3 have been shown to be regulated by IL-7 while a particular role for IL-25 has been discovered in ILC2 homeostasis and function [20, 59]. However, it remains unclear whether cytokines can specifically generate individual ILC subsets.

ILCs are able to respond to signals from the microbiota via cytokine signalling through communication with both epithelial cells and intestinal mononuclear phagocytes. However, direct interaction between ILCs and commensal microbes through TLR activation has also been shown [60]. Stimulation of human ROR*γ*t^+^ ILCs with TLR2 agonists resulted in IL-2 production, which in turn enhanced IL-22 expression, suggesting direct sensing of microbial components by ILCs occurrence. In addition, there is accumulating evidence that ILCs may directly interact with bacterial components by NCRs such as NKp46 [61]. For example, NKp46 and NKp44 have been shown to directly bind to epitopes of* Fusobacterium nucleatum* or BCG, respectively [62].

IECs were recently found to regulate the function of ILC3 cells by NF*κ*B activation [63]. An IEC specific knockout of the intrinsic inhibitor of *κ*B kinase *α* (IKK*α*), a critical regulator of NF*κ*B, led to defective ILC3 IL-22 responses, which resulted in overproduction of thymic stromal lymphopoietin (TSLP) by IECs. Notably, reductions in expression of lymphotoxin beta receptor- (LT*β*R-) dependent genes were observed in IECs from IEC specific IKK*α* knockout mice, suggesting that decreased NF*κ*Β activation downstream of LT*β*R signalling contributed to the impaired IL-22 response.

### 3.2. ILCs in Intestinal Inflammation

ILCs are able to produce large amounts of cytokines that are linked to IBD pathogenesis. In Crohn's disease patients, an increase in IL-23 responsive ILCs in the intestine was observed [64]. Also, the frequency of the ILC1 subset was much higher in inflamed intestine of people with Crohn's disease [65]. An excessive cytokine production of ILCs is likely contributing to pathogenesis, either directly acting on cells in close proximity or by the recruitment of inflammatory cells. For example, GM-CSF produced by ILCs recruits and maintains intestinal inflammatory monocytes, which can promote inflammation. It has been shown that accumulation of monocytes by GM-CSF derived from NKp46^+^ ILC3 is sufficient to control intestinal bacterial infection, even though the capacity to control inflammation is superseded by LTi-like ILC3s and T cells [66].

In general, ILCs are stimulated by cytokines and different cellular stress signals and they can respond with instantaneous cytokine production. For example, ILCs can respond to IL-1 and IL-33 released upon cellular damage and uncontrolled cell death [67, 68]. The various cytokines that different ILC subsets respond to include IL-12, IL-18, IL-1, IL-23, and TNF-family cytokine TL1A [11, 69–71]. Several studies indicate that a combination of cytokines is needed to trigger cytokine production by ILCs [72–74]. Some ILC subsets have been described to modulate adaptive immune responses via MHC class II expression and it has also been suggested that T cells may provide help to ILCs via MHC interactions [13, 75, 76]. Due to their location at the front lines of the mucosa, ILCs are able to quickly react to signals from microbiota, other immune cells in the lamina propria, and epithelial cells. Epithelial-derived signals such as IL-7 are important for the function of ILC2s and ILC3s [59]. Downregulation of epithelial-derived IL-7 expression in mice inhibited inflammation in the gastrointestinal tract of mice in a model of DSS-induced colitis [77]. Emerging studies suggest that ILCs are involved not only in the induction of inflammation but also in the maintenance of the epithelial barrier, in particular with regard to tissue repair and wound healing processes, via the different signalling pathways mentioned below.

## 4. Cross Talk between Epithelium and ILC

### 4.1. IL-22/IL-23 Pathway

IL-22 is a key cytokine for epithelial cell mediated immune responses. It is produced by several cell types but principally by ILC3s, and it has been shown to promote intestinal epithelial cell homeostasis and wound healing through STAT3 activation [45]. In addition, interaction of surface LT on ILC3s with LT*β*R on intestinal epithelial cells can lead to IL-22 production in colon in* C. rodentium* and DSS-induced colitis models [78]. The production of IL-22 by ILCs is induced by IL-23, which is associated with different models of colitis and human IBD [16, 36]. Polymorphisms of interleukin IL-23R have been linked to disease in IBD patients [79]. IL-23R is expressed by various cells, including innate immune cells such as dendritic cells and ILCs but also by epithelial cells. The work of Macho-Fernandez indicated that LT*β*R signalling in intestinal epithelial cells promotes epithelial IL-23 production in a model of DSS-induced colitis. The authors reported that epithelial-derived IL-23 promoted mucosal wound healing by inducing the IL-22-mediated proliferation and survival of epithelial cells and by increasing mucus production [80]. At steady state, the commensal microbiota stimulated IL-25 secretion by epithelial cells, which inhibited IL-22 production by ILCs [81].

Neutrophils and NK cells are additional important sources of IL-22. Neutrophils and NK cells infiltrate the colonic tissue after DSS-induced injury and produce IL-22 in an IL-23-dependent manner, thereby contributing to immune defense and restitution of epithelial integrity. The transfer of IL-22-competent neutrophils to IL-22 deficient mice even protected the colonic epithelium from DSS-induced damage [82]. Interestingly, in another study, IL-36 expression by neutrophils upon DSS-treatment preceded that of IL-22 and IL-36 shown to play a key role in epithelial barrier repair and resolution of inflammation [49]. It has been shown that maintenance of intestinal stem cells after damage is severely impaired in the absence of ILC3s or the ILC3 signature cytokine IL-22 [18]. In addition, another study found that IL-22 produced by ILCs promotes intestinal stem cell-mediated epithelial regeneration [17]. IL-22 derived from ILC3 cells increased the growth of both mouse and human small intestine organoids by increasing proliferation and promoting intestinal stem cell expansion. Moreover,* in vivo* administration of IL-22 increased epithelial regeneration and reduced intestinal pathology and mortality from induced graft-versus-host disease.

T-bet^−/−^RAG2^−/−^ (TRUC) mice develop spontaneous colitis, which is dependent on IL-23 signalling as well as the presence of neutrophils [74]. In this experimental model of IBD, IL-6 in colonic tissue augmented IL-23 and IL-1*α* secretion of most likely phagocytes, which in turn stimulated production of IL-17A and IL-22 by NCR^−^ ILC3 [39]. In addition, it has been suggested that IL23R^+^ ILCs can induce colitis via an IL-22-dependent pathway. Neutralization of IL-22 in IL-23R^−/WT^ RAG^−/−^ mice protected the mice from anti-CD40 induced acute colitis. Adding back IL-22 by hydrodynamic injections restored the pathology [15]. In this scenario, IL-22 elevated IFN-*γ* production, reduced IL-10 levels, and promoted neutrophil recruitment, which is likely the cause for the pathology seen in this model of colitis.

### 4.2. IL-22/IL-18 Pathway

IL-22 derived from ILC3s can induce IL-18 production in IECs and elevated IL-18 can amplify gut inflammation [83]. In a study by Nowarski et al., deletion of IL-18 or its receptor in intestinal epithelial cells protected mice from DSS-induced colitis and mucosal damage [84]. This group showed that IL-18 inhibited goblet cell maturation by regulating the transcriptional program instructing goblet cell development. These results suggest that goblet cell dysfunction can cause breakdown of barrier integrity and the pathology of ulcerative colitis. Another recent study showed that microbiota-associated metabolites modulated epithelial IL-18 secretion and downstream antimicrobial peptide (AMP) profiles [85]. Hyperactive IL-18 signalling induced colitis by breakdown of the mucosal barrier through induced goblet cell loss. Thus, microbiota-derived metabolites and ILCs can modulate the epithelial expression of IL-18 and thereby regulate the function of the intestinal barrier.

### 4.3. IL-36 Pathway

Recently, as noted above, it was found that IL-36R signalling is activated upon intestinal damage and stimulates IECs to drive mucosal healing [48]. IL-36 was upregulated in the colonic mucosa of patients with IBD and it was shown that, upon tissue injury, IL-36*γ* was released from IECs. Impaired proliferation of IECs and delayed wound gap closure were seen in IL-36R knockout mice and, upon mucosal damage induced by DSS, IL-36R ligands activated colonic fibroblasts that thereupon expressed GM-CSF and IL-6 and induced proliferation of IECs and their expression of the antimicrobial protein lipocalin 2.

In another study, Medina-Contreras et al. showed that IL-36R deficient mice did not recover from DSS-induced damage, which was associated with a reduction in IL-22 expression [49]. Although ILCs are potent producers of IL-22, the decrease of IL-22 expression was only seen in neutrophils. Strikingly, an aryl hydrocarbon receptor agonist increased IL-22 expression from ILC3 and CD4 T cells, which promoted full recovery from DSS-induced damage in these mice, showing the importance of IL-22 for resolution from damage. This signalling pathway could provide potential therapeutic targets to induce mucosal healing and restore epithelial integrity in IBD, especially because the expression of IL-36 is increased in patients with IBD [86].

### 4.4. TNF Pathway

Tumor necrosis factor (TNF or TNF-*α*) is a well-studied proinflammatory cytokine that is an essential mediator of inflammation in the gut. However, this cytokine is also known to cause intestinal barrier dysfunction. It is mainly produced by monocytes and macrophages and induces an increase in intestinal epithelial tight junction permeability by activating the ERK1-2 pathway [87]. The TNF superfamily of cytokines may be a promising target for IBD therapy because several members are known to play an important role in IBD pathogenesis. While blocking TNF-*α* helps many patients, it is not efficient for all patients and can also cause significant side effects, including a hampered host defense [88]. It has been shown that Gram-positive commensal bacteria are vital for the induction of DSS colitis in mice by triggering the recruitment of monocytes and macrophages, which strongly express TNF-*α* during inflammation [89]. Interestingly, a study by Roulis et al. showed that overexpression of TNF-*α* by IECs was sufficient to induce spontaneous inflammation of the terminal ileum, similar to Crohn's pathology in humans [90].

Depending on the type of stimulus, human ILC3s can switch between IL-22 and proinflammatory TNF production. For example, a combination of IL-1 and IL-23 preferentially induced IL-22 expression while NKp44 engagement with an agonistic antibody switched the cytokine profile of ILC3s to TNF. However, a combined engagement of NKp44 and the cytokine receptors for IL-1, IL-7, and IL-23 resulted in a strong synergistic effect, inducing both IL-22 and TNF [91]. TNF can also augment the IL-17 production induced by IL-23 in ILC3 cells [92].

### 4.5. IL-17 Pathway

Another cytokine that is produced by ILC3s upon intestinal inflammation and has been shown to be involved in the maintenance of barrier integrity is IL-17A [93]. During* Helicobacter hepaticus*-induced intestinal inflammation, IL-1*α* promoted the accumulation of IL-17A secreting ILCs [93]. Bacteria-driven colitis was associated with increased IL-17 and IFN-*γ* production in the colon and stimulation of colonic leukocytes with IL-23 induced IL-17 and IFN-*γ* production by innate lymphoid cells that accumulated in the inflamed colon [16]. During epithelial injury, IL-17A regulated expression of occludin, a TJ protein, and thereby limited excessive permeability of the epithelial barrier [94]. Although in this study IL-23R^+^ *γδ* T cells and not ILC3s were identified as the main producers of IL-17A in the lamina propria, it is plausible that ILCs also contribute to epithelial integrity via this pathway. In combination with TNF, IL-17 induced CXCL1, CXCL2, and CXCL5 production from intestinal epithelial cells [95, 96]. These chemokines are involved in the recruitment of neutrophils.

### 4.6. IL-33 Pathway

Until recently, ILC2 cells had not been implicated in intestinal inflammation. ILC2 cells were mainly associated with cutaneous wound healing, in an IL-33 dependent manner, and recently Monticelli et al. discovered that ILC2 cells have a role in the restoration of intestinal integrity after injury [19, 97]. The group showed a feedback loop of cytokine cues from damaged epithelium to activate innate immune cells to express growth factors essential for ILC2-dependent restoration of epithelial barrier function. This protective function of ILC2 cells seemed to be mediated by the IL-33-amphiregulin (AREG) epidermal growth factor receptor (EGFR) signalling pathway [19]. As discussed above, IECs produce IL-33 upon epithelial injury in the intestine. This study showed that, upon epithelial damage, IL-33 stimulated expression of the growth factor AREG by ILC2 cells. Genetic disruption of the endogenous AREG-EGFR pathway exacerbated disease, demonstrating a critical role for AREG-EGFR signalling in limiting inflammation. Another study by Waddell et al. showed that IL-33 can even protect against oxazolone induced colitis [98]. Because in this study it was also found that IL-33 was increased in the colon of patients with active ulcerative colitis, this suggests the IL-33 pathway has potential for the development of novel therapies.

### 4.7. IL-25 Pathway

Tuft cells, a rare cell type in the intestinal epithelium, were recently shown to promote ILC2 homeostasis and regulate IL-13 production by ILC2s through constitutive production of IL-25 [20, 99, 100]. In mice infected with* Nippostrongylus brasiliensis*, tuft cell-derived IL-25 increased IL-13 production by ILC2s, which in turn led to increased frequencies of tuft and goblet cells supporting helminth clearance [20]. These results indicate a role for tuft cell-derived IL-25 in the physiological host response to helminths and the maintenance of ILC2s in intestinal epithelium. While this feedback loop between tuft cells and ILC2s is not directly implicated in IBD, ILC2s have recently been suggested to play a role in wound healing in lung mucosa and may very well have a similar function in wound healing in the intestinal mucosa [101]. However, further research is needed to clarify the role of ILC2s in mucosal damage of the intestine.

### 4.8. IFN-*γ* Pathway

Bacteria-driven and anti-CD40 induced innate colitis are both associated with an increased production of IL-17 and IFN-*γ* in the colon, shown by Buonocore et al. [16]. Upon stimulation of colonic leukocytes with IL-23, IL-17 and IFN-*γ* were produced exclusively by ILCs that accumulated in the inflamed colon [16]. IFN-*γ* can be produced by both group 1 ILCs and group 3 ILCs.

After injury of the intestinal epithelium, enterocytes surrounding damaged epithelium can migrate into the lesion in response to various cytokines to initiate wound healing. It has been shown that IFN-*γ* inhibits enterocyte migration by preventing interenterocyte gap junction communication [102]. Furthermore, IFN-*γ* has also been identified as an IBD susceptibility gene locus by GWAS, supporting the inflammatory role of this cytokine [50]. In contrast, previous work from Dignass and Podolsky showed that TGF*α*, EGF, IL-1*β*, and IFN-*γ* enhanced epithelial cell restitution by 2.3-fold to 5.5-fold [42]. More recently, Muzaki et al. showed that IFN-*γ* can trigger an early anti-inflammatory response in intestinal epithelial cells, induced by a particular DC subset [103]. Interestingly, in an innate colitis model in which RAG^−/−^ mice were treated with anti-CD40 antibody and IL-22 was found to be pathogenic, the group of Eken et al. showed a significant reduction of IFN-*γ* levels in the colon after IL-22 neutralization during colitis [15]. As IL-22 was induced in ILC3 by IL-23 signalling, these data suggest that IFN-*γ* may contribute to IL-23-IL-23R-dependent colitis. A crucial role for IFN-*γ* in promoting colitis is further corroborated by Uhlig et al., where the authors abrogate the inflammatory response by blocking IFN-*γ* [104]. However, the exact role of ILC derived IFN-*γ* in maintaining epithelial barrier integrity and mucosal inflammation remains to be elucidated.

### 4.9. ILC Depletion

ILC depletion strategies, using anti-Thy1 or anti-CD90 monoclonal antibodies, have been successful in two different experimental models of IBD,* Helicobacter hepaticus*-induced intestinal inflammation and T-bet^−/−^Rag2^−/−^ (TRUC) mice that develop spontaneous colitis, respectively [16, 39]. In contrast, there is also evidence that ILC depletion is not beneficial for intestinal homeostasis. A study by Hepworth et al. showed that depletion of MHC class II^+^ ILC3s dysregulated CD4^+^ T cell responses and promoted spontaneous colitis [105].

So far, ILC depletion is only seen in Simian Immunodeficiency Virus infected macaques and HIV infected humans [106, 107]. Similar to IBD, HIV infection can induce breakdown of the intestinal epithelial barrier. Damage of the mucosal epithelium is preceded by acute HIV infection, which leads to rapid depletion of gastrointestinal IL-17 and IL-22 producing CD4^+^ T cells [108]. Because ILCs, not CD4^+^ T cells, are the most rapid producers of IL-17 and IL-22, Kløverpris et al. investigated the role of ILCs in gut epithelial repair during HIV infection [35, 107, 109]. It was shown that ILCs were depleted from the blood during early acute HIV-1 infection and ILC numbers did not recover after resolution of peak viremia. ILC depletion was associated with upregulation of genes associated with cell death and apoptosis and coincided with epithelial barrier breakdown. During the chronic phase of infection, ILC depletion was associated with altered subset composition and increased expression of activation, tissue homing markers, and Fas death receptor. Tonsil- and gut-resident ILCs were not enriched or depleted in chronic HIV-1 infection, suggesting that the depletion of circulating ILCs was due to apoptosis instead of redistribution. These data suggest that ILC apoptosis could be associated with the loss of epithelial barrier integrity.

## 5. Conclusion

To date, there is no cure for IBD and current treatments focus on blocking the inflammatory response, which can cause significant side effects, including a hampered host defense. Consequently, new therapeutic approaches particularly focus on target molecules with a more limited range of functions, which also should have less side effects. The feedback loop in which damaged epithelium activates ILCs to restore epithelial barrier function is of interest for future therapeutic approaches. One important feedback loop is the IL-22/IL-23 pathway, where IL-23 derived from activated IECs induces the production of IL-22 from ILCs. IL-22 produced by ILC3s promotes intestinal stem cell-mediated epithelial regeneration and wound healing. Recently, it was found that IL-36R signalling stimulates IECs upon intestinal damage and drives mucosal healing in an IL-22 dependent manner. Besides its role in mucosal healing, IL-22 derived from ILC3s can also induce IL-18 production in IECs and thereby amplify gut inflammation. Dependent on the type of stimulus, ILC3s can switch between IL-22 and TNF production. TNF favors IEC apoptosis and correspondingly augments the IL-17 production induced by IL-23 in ILC3s, a mechanism that is also involved in the maintenance of barrier integrity. In addition, ILC2s are involved in barrier integrity by the IL-33-AREG-EGFR signalling pathway. The different signalling pathways between damaged intestinal epithelium and ILCs indicate that feedback loops between these cell types are critical for maintaining epithelial barrier integrity and tissue homeostasis.
